# Sex-Specific Regulation of Mitochondrial DNA Levels: Genome-Wide Linkage Analysis to Identify Quantitative Trait Loci

**DOI:** 10.1371/journal.pone.0042711

**Published:** 2012-08-20

**Authors:** Sonia López, Alfonso Buil, Juan Carlos Souto, Jordi Casademont, John Blangero, Angel Martinez-Perez, Jordi Fontcuberta, Mark Lathrop, Laura Almasy, Jose Manuel Soria

**Affiliations:** 1 Unit of Genomic of Complex Diseases, Institute of Biomedical Research of Hospital de la Santa Creu i Sant Pau, Barcelona, Spain; 2 Haemostasis and Thrombosis Unit, Department of Haematology, Hospital de la Santa Creu i Sant Pau, Universitat Autònoma de Barcelona, Barcelona, Spain; 3 Internal Medicine Department, Hospital de la Santa Creu i Sant Pau, Universitat Autònoma de Barcelona, Barcelona, Spain; 4 Department of Population Genetics, Texas Biomedical Research Institute, San Antonio, Texas, United States of America; 5 Institut de Génomique, Centre National de Génotypage, Evry, France; University of Texas Health Science Center at San Antonio, United States of America

## Abstract

Altered mitochondrial DNA (mtDNA) levels have been associated with common diseases in humans. We investigated the genetic mechanism that controls mtDNA levels using genome-wide linkage analyses in families from the Genetic Analysis of Idiopathic Thrombophilia Project (GAIT). We measure mtDNA levels by quantitative real-time PCR in 386 subjects from 21 extended Spanish families. A variance component linkage method using 485 microsatellites was conducted to evaluate linkage and to detect quantitative trait loci (QTLs) involved in the control of mtDNA levels. The heritalibility of mtDNA levels was 0.33 (p = 1.82e-05). We identified a QTL on Chromosome 2 (LOD = 2.21) using all of the subjects, independently on their sex. When females and males were analysed separately, three QTLs were identified. Females showed the same QTL on Chromosome 2 (LOD = 3.09), indicating that the QTL identified in the analysis using all of the subjects was a strong female QTL, and another one on Chromosome 3 (LOD = 2.67), whereas in males a QTL was identified on Chromosome 1 (LOD = 2.81). These QTLs were fine-mapped to find associations with mtDNA levels. The most significant SNP association was for the rs10888838 on Chromosome 1 in males. This SNP mapped to the gene *MRPL37*, involved in mitochondrial protein translation. The rs2140855 on Chromosome 2 showed association in the analysis using all of the subjects. It was near the gene *CMPK2*, which encodes a mitochondrial enzyme of the salvage pathway of deoxyribonucleotide synthesis. Our results provide evidence of a sex-specific genetic mechanism for the control of mtDNA levels and provide a framework to identify new genes that influence mtDNA levels.

## Introduction

Mitochondria are the only cellular organelles (apart from the nucleus) that contain a genome, the mitochondrial DNA (mtDNA). It is inherited exclusively through the maternal line. Human mtDNA consist of one circular double-stranded DNA molecule of 16569 bp, which presents different characteristics compared with the nuclear genome. It is about 0.5–1% of the total DNA human cell content. A variable number of mtDNA molecules are located within the mitochondrial matrix, attached to the inner mitochondrial membrane near the oxidative phosphorilation (OXPHOS) system. The mitochondrial genome encodes few, but essential proteins of the OXPHOS system (13 polypeptides, 22 tRNA and 2 rRNA). Then, any defect of mtDNA can lead finally to mitochondrial dysfunction and energetic cell impairment. Thus, mitochondria play a pivotal role in cell life and death in almost all eukaryotic cells, as they are vital organelles involved in cellular energy production and apoptosis [1]. However, most of the mitochondrial proteins are encoded by the nuclear DNA and synthesized into the cytoplasm, from which they are imported to mitochondria. Consequently, the mitochondrial biogenesis depends on the coordinated expression of both nuclear and mitochondrial genomes.

Mitochondria are considered the major source of reactive oxygen species (ROS) and mtDNA is one of the main targets of oxidative stress. During the last years, a large number of clinical disorders have been linked to mitochondrial dysfunction where oxidative stress was the main pathophysiologic underlying mechanism. In addition, sequence variation in mtDNA contributes to the risk of disease and it has been associated with serious human disorders [2], [3], [4]. Also, sequence variation of the mtDNA may be linked to functional differences and thereby lead to different oxidative phenotypes [5]. Interestingly, the variation of appropriate mtDNA levels has been linked also to a variety of common disorders [6], [7], [8]. In addition, it has been reported that altered mtDNA levels correlates with malignancy [9], [10], [11] and even with prognosis [12], [13]. Moreover, there are several reports that attempt to establish mtDNA quantity as an oxidative stress biomarker to predict the risk for cancer [14], [15].

Some of the mitochondria-associated disorders include obesity, cancer, diabetes, neurodegenerative diseases, sepsis, ischemia/reperfusion injury as well as the physiologic process of ageing [16], [17], [18]. In addition, there is also increasing evidence supporting that oxidative stress plays a key role in the development of cardiovascular diseases and atherosclerosis [19], [20], [21]. As a consequence, there is a growing interest in describing the mechanisms that regulate the mitochondrial biogenesis. Particularly, much effort is being put towards increasing our knowledge about the maintenance of the mitochondrial genome, since both the integrity and quantity of mtDNA are critical for maintaining mitochondrial respiratory capacity to reach the ATP levels necessary for cell viability.

Despite the identification of a number of nuclear DNA-encoded trans-acting factors that regulate mitochondrial biogenesis, the exact biological mechanisms responsible for the control of mtDNA levels are unknown. The aim of our study was to identify genes that influence the variation of mtDNA levels and to determine if sex modulates the genetic mechanism that controls these levels. For this purpose, we conducted genome-wide linkage analyses in families from the Genetic Analysis of Idiopathic Thrombophilia Project (GAIT). In addition, we fine-maped the genetic determinants detected by the linkage analyses.

## Methods

### Subjects

Our study included 386 subjects belonging to 21 Spanish families from the GAIT Project. An extensive description of the GAIT Project has been published previously [22]. Each family consisted of 3 to 5 generations of subjects that ranged in age from 0.34 to 87.9 years, with a mean of 37.6 years. There were a similar number of males and females in our study. Twelve families were selected through a proband with idiopathic thrombophilia and the remaining 9 families were randomly selected without regard to phenotype. Thrombophilia was defined as recurrent thrombotic episodes (at least one of which was spontaneous) a single spontaneous thrombotic event with a first-degree relative also affected, or early-onset thrombosis (≤45 years). The proband's thrombophilia was considered idiopathic when all known biological causes of thrombophilia (i.e. Protein S and Protein C deficiencies, antithrombin deficiency, activated protein C resistance, plasminogen deficiency) at the time of recruitment were excluded. Subjects that had a personal or familiar history suggestive of mitochondrial disease or neuromuscular disorders were excluded.

### Ethics Statement

The Institutional Review Board of the Hospital de la Santa Creu i Sant Pau (Barcelona) approved all protocols of the study. Adult subjects gave informed consent for themselves and for their minor children.

### Blood Collection and DNA Extraction

Blood was collected by venipuncture in Vacutainer® Citrate tubes from fasting subjects. Thrombophilic participants were not using oral anticoagulants at the time of sampling.

DNA was extracted by a standard salting-out procedure from the buffy coat [23] and used for quantifying and genotyping the mtDNA.

### Genotyping with Microsatellites and SNPs

All subjects were genotyped for a genome-wide scan including 485 microsatellite markers distributed through the autosomal genome at an average interval of 7.1 cM. The average heterozygosity of the microsatellite markers was 0.79. Microsatellites consisted primarily of the ABI Prism Linkage Mapping Set MD-10 (Applied Biosystems, Foster City, CA). Linkage mapping was undertaken with the PE LMS II fluorescent marker set (ABI Prism, Foster City, CA) with multiplex polymerase chain reaction (PCR) [24]. The products from the PCR were analyzed on the PE 310, PE 377, and PE 3700 automated sequencers, and genotyped using the Genotyper software. Information on microsatellite markers can be found in the public-accessible genomic database (http://www.gdb.org). Marker maps for multipoint analyses were obtained from the Marshfield Medical Research Organization (http://research.marshfieldclinic.org/genetics/).

Fine-mapping of linkage regions was carried out using 4,448 SNPs from the Illumina platform (San Diego, CA, USA). The SNPs with a genotype call rate<0.95, a minor allele frequency <0.025 or failed the Hardy-Weinberg equilibrium (HWE) test (p<5e-7) were excluded from the analysis. HWE was ascertained by a standard χ^2^ with 1 degree of freedom and was tested using parental data only.

The program INFER (PEDSYS) [25] was used to check for Mendelian inconsistencies in the genotypic data. Mistypings and markers for discrepant subjects were either corrected or excluded from the analyses.

### Phenotyping: Quantitative Analysis of mtDNA

Quantitative real-time PCR (ABI Prism 7000 Sequence Detector System, Applied Biosystems, Foster City, CA) was used to determine mtDNA levels in total DNA from the buffy coat of each GAIT individual. For each DNA extract, the highly conserved mitochondrial *ND2* gene (mtDNA) and the housekeeping *18S rRNA* nuclear gene (nDNA) were quantified separately (Power SYBR® Green PCR Master Mix, Applied Biosystems, Foster City, CA). A double-stranded DNA dye (SYBR Green I) was used to monitor product formation continuously [26] and ROX dye was used as reference.

The PCR amplification of a 235 bp fragment length of the *ND2* gene was performed by using the forward 5′-GCCCTAGAAATAAACATGCTA-3′ and the reverse 5′-GGGCTATTCCTAGTTTTATT-3′ primers. For the *18S rRNA* gene, the forward 5′-ACGGACCAGAGCGAAAGCAT-3′ and the reverse 5′-GGACATCTAAGGGCATCACAGAC-3′ primers were used for the amplification of a 531 bp fragment length.

#### PCR conditions for the mitochondrial gene

The PCR reactions for the amplification of the *ND2* gene contained 250 nM of each primer, 10 µl of the Power SYBR® Green PCR Master Mix (2×) and 2 ng of the DNA extract in 20 µl volume. The ABI Prism absolute quantification programme conditions for *ND2* consisted of a single denaturation-enzyme-activation of 10 min at 95°C, followed by 35 cycles of amplification. Each cycle consisted of a denaturation step of 15 sec at 95°C, an annealing step of 20 sec at 53°C and an extension step of 30 sec at 72°C.

#### PCR conditions for the nuclear gene

The PCR reactions for the amplification of the *18S rRNA* gene contained 300 nM of each primer, 10 µl of the Power SYBR® Green PCR Master Mix (2×) and 2 ng of the DNA extract in 20 µl volume. The ABI Prism absolute quantification programme conditions for *18S rRNA* consisted of a single denaturation-enzyme-activation of 10 min at 95°C, followed by 37 cycles of amplification. Each cycle consisted of a denaturation step of 15 sec at 95°C, an annealing step of 20 sec at 66°C and an extension step of 40 sec at 72°C.

After the amplification of each gene, a dissociation curve analysis was carried out to determine the specificity of the PCR product by the melting temperature [27].

A standard curve for each gene was always included in each PCR run by amplifying serial dilutions of a control human genomic DNA (TaqMan® Control Genomic DNA (Human; 10 ng/ul), Applied Biosystems, Foster City, CA). The quantity (pg) of each target sequence (mitochondrial and nuclear) was calculated from the corresponding standard curve. Two nanograms of the control human genomic DNA was also added in each PCR run as a positive control. As a negative control, the template DNA was replaced with PCR-grade water. The results of the mtDNA levels were expressed as mtDNA quantity to nuclear DNA quantity ratio (mtDNA/nDNA) [28], [29].

### Linkage and Association Analyses

A standard multipoint variance component linkage method was used to assess linkage between autosomal markers and mtDNA levels using the Sequential Oligogenic Linkage Analysis Routines (SOLAR) v. 4.0 software package [30]. Age, sex, smoking behaviour, oral contraceptives use and the household effect were included in the variance component framework as linear predictors of the phenotype. Previous studies suggested that such a method might be vulnerable to deviations from multivariate normality and particularly to high levels of kurtosis in the trait's distribution, giving inflated logarithm of odds (LOD) scores [31]. Levels of mtDNA in the GAIT sample exhibited a kurtosis of 0.57, which does not affect the distribution of LOD scores. Thus, the standard nominal p-values for LOD scores are appropiate for mtDNA linkage screen [32]. Moreover, as 12 of the GAIT families were ascertained through thrombophilic probands, all analyses included an ascertainment correction achieved by conditioning the likelihood of these pedigrees on the likelihoods of their respective probands [33]. Finally, to control the multiple testing effect, genome-wide p-values were calculated using the method of Feingold et al. [34].

The association between SNPs and mtDNA levels was tested using a measured genotype association analysis assuming an additive model of allelic effect [35]. The p-values for each SNP association test were evaluated under two different approaches of correction for multiple testing. First, we applied the generally more stringent Bonferroni correction, which establishes a significance threshold corresponding to a family-wise error rate of 0.05. Second, the more lenient Benjamini-Hochberg (B-H) adjustment was also applied to the p-values, assuming a 10% false discovery rate [36].

To investigate the potential influence of sex on the genetic mechanism of mtDNA levels, we performed both genome-wide linkage and SNP association analyses based on the whole sample, on females only and on males only.

## Results

### Phenotypic Data and Heritability of mtDNA

Table 1 summarises the phenotypic data of all of the subjects in our study. A similar number of males and females were included in the analysis. No significant differences were found between the two sexes with regard to age, but not with regard to smoking behaviour (p = 0.0022). Oral contraceptives use was the only covariate that showed a significant effect on mtDNA levels (p = 0.0072). The estimated proportion of mtDNA levels variance due to oral contraceptives use that was included in the analysis was 1.57%, and its effects were estimated simultaneously with the genetic effects. We found no significant differences in mtDNA levels between males and females (0.25±0.11 and 0.24±0.11, respectively; p = NS). Genetic heritability (h^2^) of mtDNA levels was 0.33±0.09 (p = 1.82e-5), indicating that 33% of the phenotypic variation in this trait is due to the additive effect of genes.

**Table 1:** Phenotypic Characteristics of the 386 Subjects Included in the GAIT Project.

Phenotypic characteristics	Phenotypic data			P-value*
Number of families	21			
	All subjects	Males	Females	
Number of subjects, n (%)	386 (100)	173 (45)	213 (55)	NS
Median age, years ± SD	37.6±19.94	38.3±19.99	36.7±19.91	NS
Current smokers†, n (%)	144 (37)	79 (46)	65 (31)	0.0022
Oral contraceptives‡, n (%)	15 (4)	—	15 (7)	—
mtDNA levels§, ratio ± SD	0.24±0.11	0.25±0.11	0.24±0.11	NS

† Subjects in the study were defined as currently smokers when they smoke independently of the number of cigarettes.

‡ Oral contraceptives use at inclusion.

§ mtDNA levels were expressed as the mtDNA to nuclear DNA ratio (mtDNA/nDNA).

SD: standard deviation.

NS: not significant.

* P-value<0.05 was considered statistically significant.

### Autosomal QTLs Influencing mtDNA Levels

**Figure 1:**
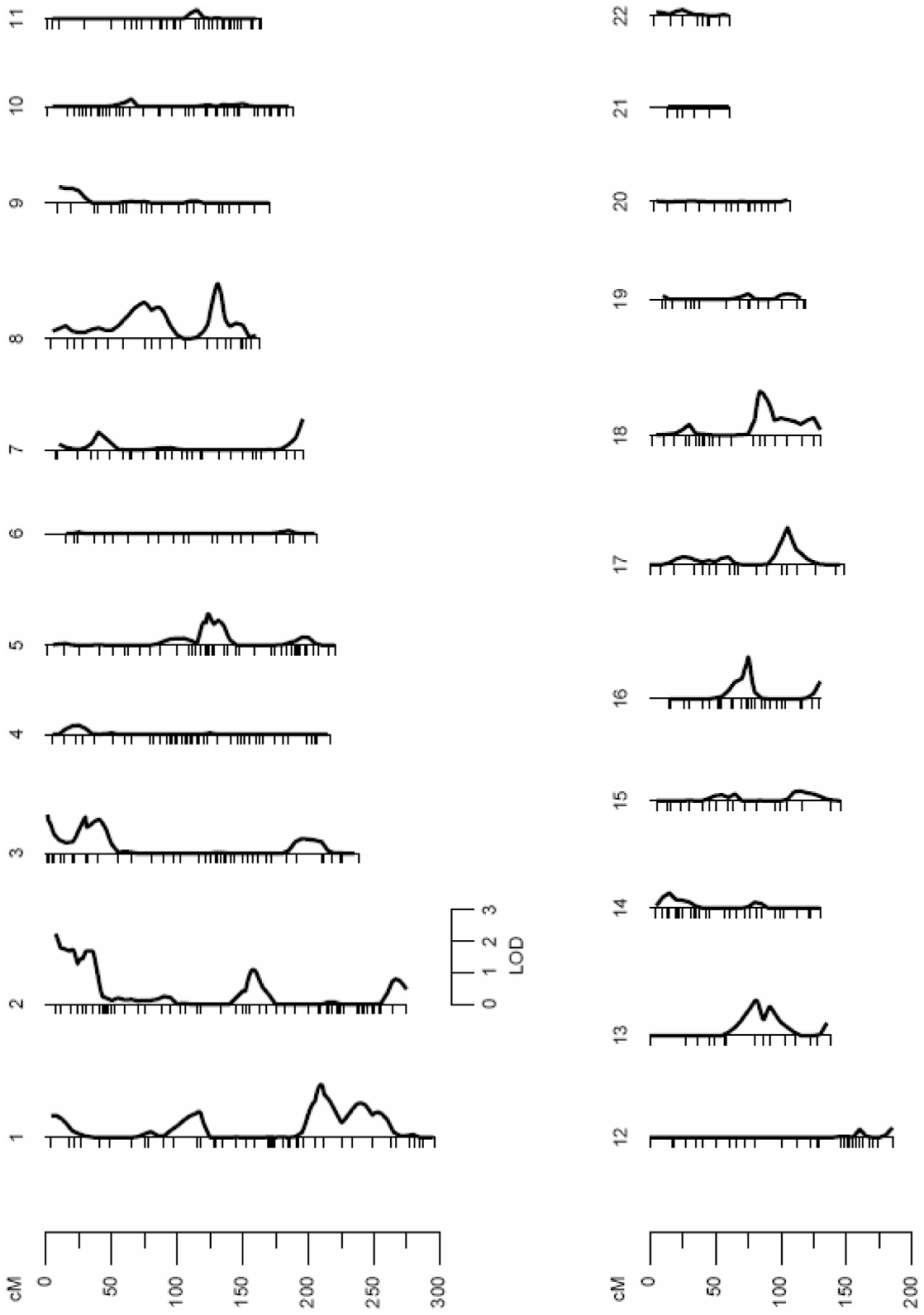
Linkage analysis for mtDNA levels in subjects of both sexes. There is a linkage signal in Chromosome 2 with a peak LOD score of 2.21 (p = 7.09e-04) defining a quantitative trait locus for mtDNA levels in a region that maps to 2p25.3-2p24.3.

**Figure 2:**
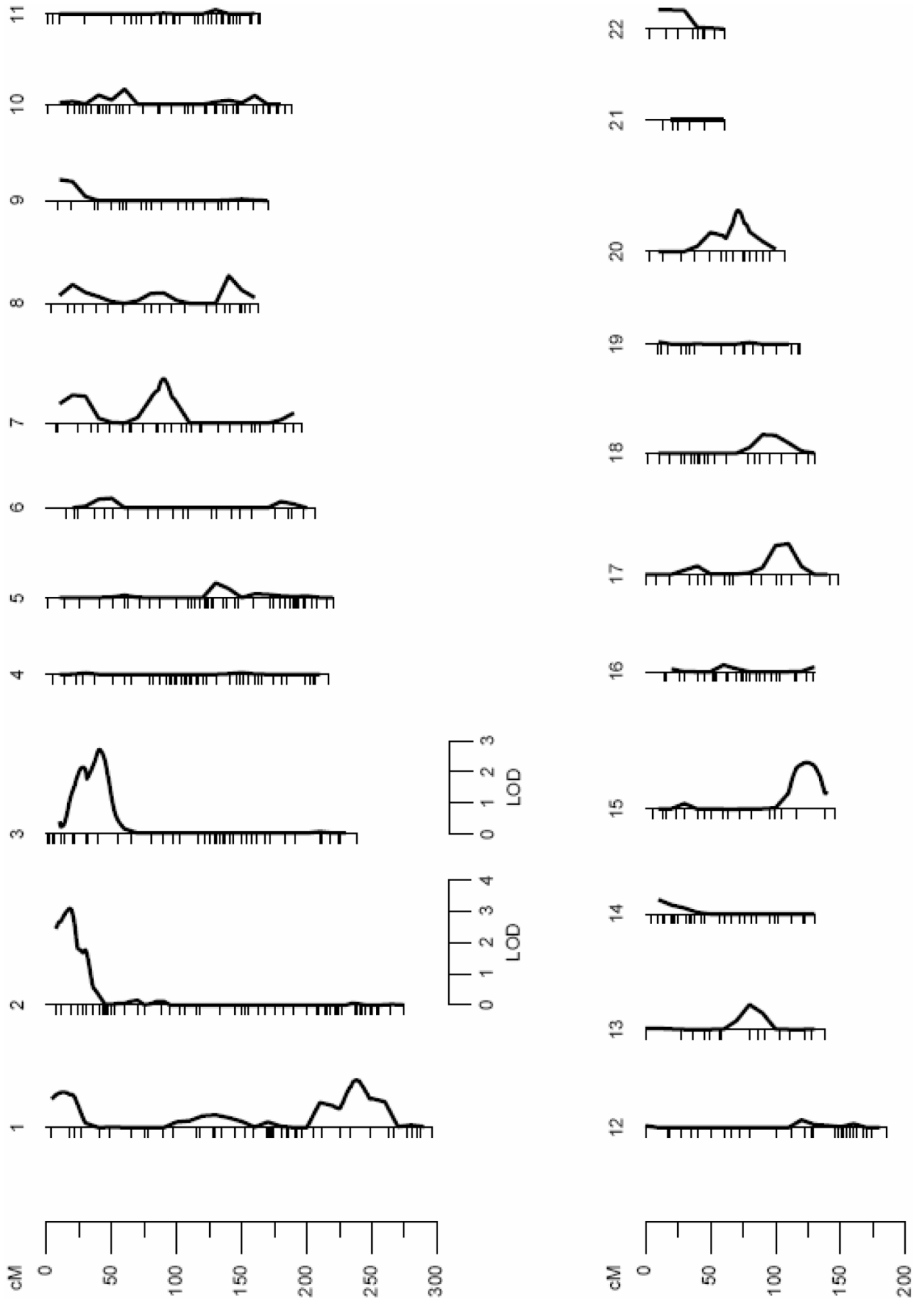
Linkage analysis for mtDNA levels in females only. The linkage signal detected in Chromosome 2 in the first analysis including the subjects of both sexes remains in the analysis performed with females only. However, this linkage signal in the sex-specfic analysis becomes better defined and more significant (LOD score of 3.09; p = 8.11e-05). In addition, a new quantitative trait locus for mtDNA levels is detected in Chromosome 3 only in females (LOD score of 2.67; p = 2.27e-04). Fine-mapping of the female-specific QTLs detected on Chromosome 2 and Chromosome 3 was carried with a set of 790 and 2687 SNPs, respectively.

**Figure 3:**
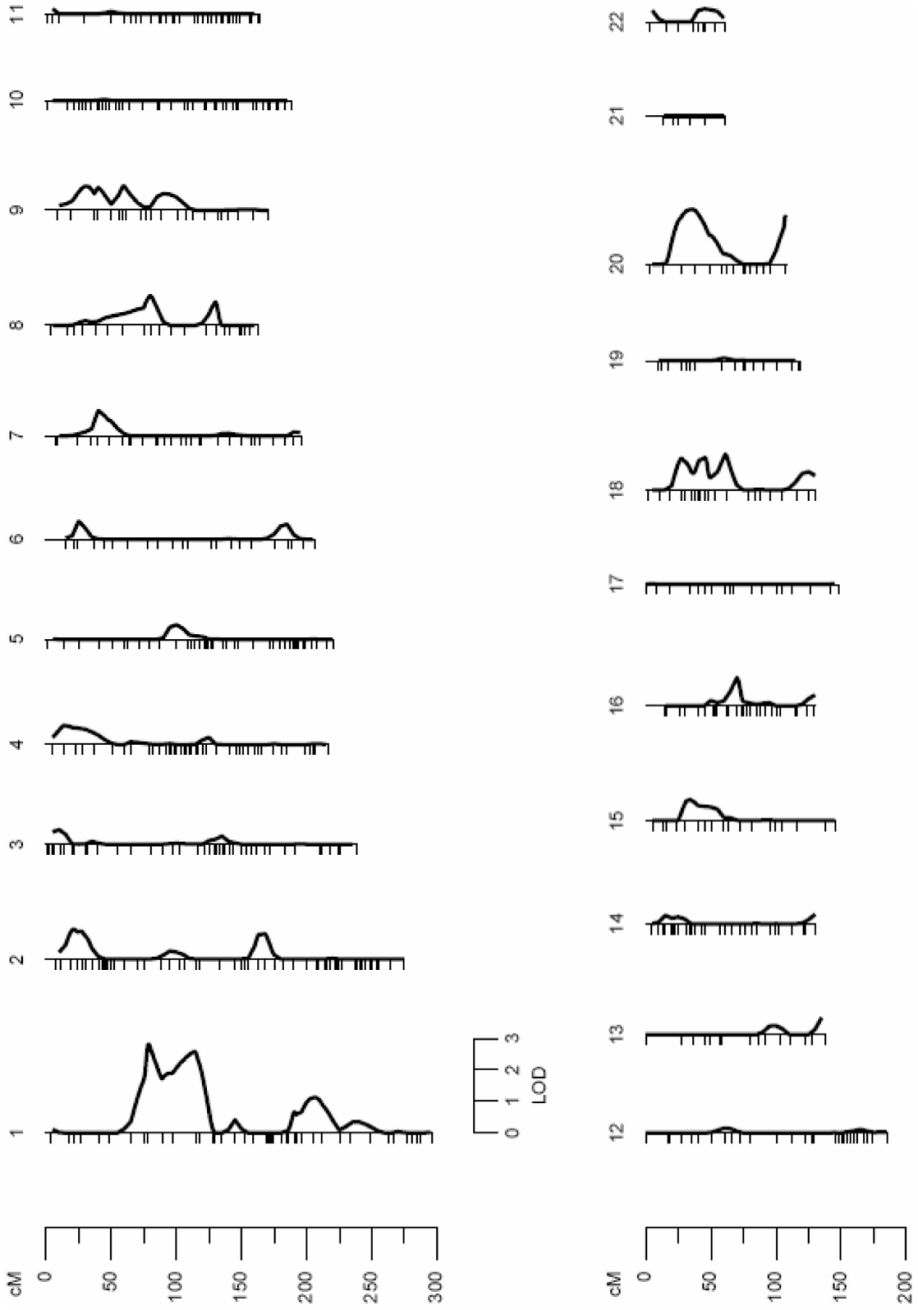
Linkage analysis for mtDNA levels in males only. The linkage signal detected in Chromosome 2 in the first analysis including the subjects of both sexes completely disappears in the analysis performed with males only. However, a new significant linkage signal for mtDNA levels is detected in Chromosome 1 only in males (LOD score of 2.81). Fine-mapping in this linkage region with 971 SNPs reveals the most significant SNP association with mtDNA levels for the rs10888838 (MAF = 0.1133; p = 4.01e-06) in the analysis with males only. This SNP was located in the gene *MRPL37*, which emerges as a strong candidate gene for the control of mtDNA levels in males.

A standard multipoint variance-component method was used to assess linkage between microsatellites and mtDNA levels. A first linkage analysis was carried out using all of the subjects. The results revealed a suggestive quantitative trait locus (QTL) on the short arm of Chromosome 2 (2p), which may influence the variation of mtDNA levels (LOD score = 2.21; p = 7.09e-04) (Figure 1). The QTL on Chromosome 2 was detected through a peak LOD score located between 3137230 bp and 16522359 bp, in a region that maps to 2p25.3-2p24.3. Specific data from the linkage analysis are shown in Table 2. Further genetic linkage analyses were conducted separating the subjects according to their sex to detect sex-specific genomic regions that may regulate the variation of mtDNA levels. The linkage analysis performed with females only revealed two QTLs (Figure 2). One significant linkage signal was detected on Chromosome 2 (LOD score = 3.09; p = 8.11e-05), at the same genomic region (2p25.3-2p25.1) that the signal previously detected in the analysis with the subjects of both sexes. This signal defined a QTL for mtDNA levels that comprises a region between 3137230 bp and 8605209 bp on Chromosome 2 (Table 2). Another linkage region was detected in females on the short arm of Chromosome 3 (3p) with a LOD score of 2.67 (p = 2.27e-04) (Figure 2). This linkage signal was located between 7544027 bp and 23223909 bp, in a region that maps to 3p26.2-3p24.2 (Table 2). Interestingly, the results from the linkage analysis performed in males only differed from those obtained in females. The linkage signal previously detected on Chromosome 2 completely disappeared in males. However, a new genomic region was detected on the short arm of Chromosome 1 (1p) (LOD score = 2.81; p = 1.57e-04), suggesting a sex-specific genetic mechanism for the regulation of mtDNA levels (Figure 3). The male-specific QTL was detected through a peak LOD score located between 45992690 bp and 56878848 bp, in a region that maps to 1p34.1-1p32.2 (Table 2).

**Table 2:** Data of the Linkage Analysis for mtDNA Levels in GAIT.

		All subjects		Males		Females	
Linkage signal	Chr. Position	LOD score	Genome-wide P-value	LOD score	Genome-wide P-value	LOD score	Genome-wide P-value
Chr. 2	2p25.3-2p24.3*	2.21	7.09e-04	—	—	3.09	8.11e-05
Chr. 3	3p26.2-3p24.2	—	—	—	—	2.67	2.27e-04
Chr. 1	1p34.1-1p32.2	—	—	2.81	1.57e-04	—	—

Chr.: Chromosome.

* This Chromosome position refers to the linkage signal from the analysis using all of the subjects. Chromosome position for the linkage signal detected in Chromosome 2 only in females is 2p25.3-2p25.1. All Chromosome positions were based on the National Center for Biotechnology Information (NCBI) build 36.

A bioinformatic search in these linkage regions showed several potential candidate genes for the sex-specific control of mtDNA levels. A brief summary of these data can be found in Table 3. Broad data regarding biological function of each candidate gene and their potential role in disease have been compiled in a supplementary table (Table S1).

**Table 3:** Candidate Genes Proposed for Sex-specific Variation of mtDNA Levels in GAIT.

Gene symbol	Description	Chr. Position	Sex-specificity	Subcellular location	Biological process
*CMPK2*	*cytidine monophosphate (UMP-CMP) kinase 2*	2p25.2	Females	Mitochondrion	Pyrimidine biosynthesis of the salvage pathway
*RNASEH1*	*ribonuclease H1*	2p25.3	Females	Nucleus Mitochondrion	RNA degradation during DNA replication
*MRPS25*	*mitochondrial 28S ribosomal protein S25*	3p25.1	Females	Mitochondrion	Mitochondrial translation
*TIMM40*	*coiled-coil-helix-coiled-coil-helix domain containing 4 (CHCHD4)*	3p25.1	Females	Mitochondrion	Protein transport from the intermembrane space to the mitochondrial matrix
*C3orf31*	*chromosome 3 open reading frame 31 (MMP37-like protein)*	3p25.2	Females	Mitochondrion	Protein import into mitochondrial matrix
*PPARG*	*peroxisome proliferator-activated receptor gamma*	3p25.2	Females	Nucleus	Nuclear transcription
*OGG1*	*8-oxoguanine DNA glycosylase*	3p25.3	Females	Nucleus Mitochondrion	DNA repair
*MRPL37*	*mitochondrial 39S ribosomal protein L37*	1p32.3	Males	Mitochondrion	Mitochondrial translation
*PARS2*	*prolyl-tRNA synthetase 2*	1p32.3	Males	Mitochondrion	Prolyl-tRNA aminoacylation during protein biosynthesis
*ATPAF1*	*ATP synthase mitochondrial F1 complex assembly factor 1*	1p33	Males	Mitochondrion	Protein complex assembly
*CPT2*	*carnitine palmitoyltransferase 2*	1p32.3	Males	Mitochondrion	Fatty acid metabolism, β-oxidation
*UQCRH*	*ubiquinol-cytochrome c reductase hinge protein*	1p34.1	Males	Mitochondrion	Mitochondrial electron transport
*CMPK1*	*cytidine monophosphate (UMP-CMP) kinase 1*	1p33	Males	Cytoplasm	Pyrimidine ribonucleotide biosynthesis

Chr.: Chromosome.

### Fine-mapping of the Sex-specific QTLs

We conducted a specific search for genotype-phenotype associations in the sex-specific QTLs detected in our genome-wide linkage analyses to detect common variants susceptible to affect mtDNA levels. The most significant SNPs with their p-values included in these genomic regions are shown in Table 4.

**Table 4:** Top Significant SNP-associations with mtDNA Levels in GAIT.

Gene	SNP	Chr	Sex-specificity	MAF†	beta‡	P-value*
*MRPL37*	rs10888838	1	Males	0.1133	0.806	4.01e-06(a)
*RNF144*	rs2140855	2	No	0.3879	0.321	1.85e-05(a)
*RNF144*	rs2056634	2	No	0.1928	0.344	3.50E-04(b)
*RNF144*	rs11682769	2	No	0.2819	−0.275	4.90E-04(b)
*FLJ41046*	rs2609101	2	No	0.2282	0.317	2.14E-04(b)
*FLJ41046*	rs2564023	2	No	0.0692	−0.454	3.40E-04(b)
*FLJ41046*	rs1880800	2	No	0.4739	−0.248	3.81e-04(b)
*FLJ41046*	rs7571496	2	No	0.3032	0.241	9.27E-04(b)
*SOX11*	rs813779	2	No	0.2315	−0.323	2.97E-04(b)
*TSSC1*	rs2305491	2	No	0.0805	0.447	4.91e-04(b)
*VENTXP7*	rs2731943	3	Females	0.2541	0.489	6.93e-05(b)
*ZNF659*	rs161196	3	No	0.0301	0.903	6.94E-05(b)
*SATB1*	rs7635386	3	Females	0.3835	0.385	9.47e-05(b)

Chr.: Chromosome.

† Minimal Allele Frequency of the SNP in our sample.

‡ represents the effect of one copy of the rare allele in mtDNA levels when it is expressed in original scale.

* P-value of the association with mtDNA levels.

(a) indicates statistical significance after Bonferroni correction for multiple testing.

(b) indicates statistical significance after B-H adjustment for multiple testing assuming a 10% of false discovery rate.

#### Fine-mapping of the male-specific QTL on Chromosome 1

A total of 971 SNPs were typed in the linkage region detected on Chromosome 1 in males. Among them, the rs10888838 (p = 4.01e-06) remained significant for the association with mtDNA levels after applying the Bonferroni correction for multiple testing (Table 4). This SNP association was significant in the analysis with males, but not with females (data not shown). The rs10888838 is located in the gene *mitochondrial 39S ribosomal protein L37* (*MRPL37*) on Chromosome 1p32.3. We want to emphasise that *MRPL37* was identified by linkage and association analyses. Such genetic convergence, obtained by two different analytical strategies, gives more confidence in our results.

#### Fine-mapping of the female-specific QTLs on Chromosome 2 and Chromosome 3

A total of 790 SNPs were genotyped to fine-map the linkage region detected on Chromosome 2. Among these SNPs, the rs2140855 (p = 1.85e-05) remained significant after applying the Bonferroni correction. This intergenic polymorphism is located on Chromosome 2p25.2 at ∼170 Kbp downstream of the gene *RNF144* (*ring finger protein 144A*) and ∼380 Kbp downstream of the gene *cytidine monophosphate (UMP-CMP) kinase 2* (*CMPK2*). In addition, other 8 SNPs in this QTL showed significant association with mtDNA levels after applying the B-H adjustment assuming 10% false discovery rate (Table 4). Notably, 2 of these SNPs (rs2056634, p = 3.50e-04 and rs11682769, p = 4.90e-04) are at the same locus as rs2140855, and also around *RNF144* and *CMPK2*. Moreover, 4 of these polymorphisms (rs2609101, p = 2.14e-04; rs2564023, p = 3.40e-04; rs1880800, p = 3.81e-04 and rs7571496, p = 9.27e-04) are in or around *FLJ41046*, at a distance of ∼700 Kbp upstream of the gene *CMPK2*. The rs813779 (p = 2.97e-04) and rs2305491 (p = 4.91e-04) were the remaining two SNPs that passed the B-H adjustment with 10% false discovery rate. They are around the gene *Transcription factor SOX-11* (*SOX11*) and in the gene *tumor suppressing subtransferable candidate 1* (*TSSC1*) respectively. Although all these SNPs are located in the female-specific QTL, the association with the phenotype only reached statistical significance in the analysis using all of the subjects.

A total of 2687 SNPs were genotyped to fine-map the linkage region detected on Chromosome 3 in females. None of the analysed SNPs remained significant after applying the Bonferroni correction. However, three SNPs reached statistical significance when applied the B-H adjustment assuming 10% false discovery rate (Table 4). These SNPs were rs2731943 (p = 6.93e-05) located around *VENTXP7* (*VENT homeobox (Xenopus laevis) pseudogene 7*) rs7635386 (p = 9.47e-05) located in *SABT1* (*special AT-rich sequence binding protein 1*) and rs161196 (p = 6.94e-05) located in *ZNF659* (*zinc finger protein 385D*). Despite that *ZNF659* is located in a female-specific QTL, the rs161196 in this gene showed significant association with mtDNA levels only in the analysis with all of the subjects.

## Discussion

Genetic variation and altered levels of mtDNA have been associated with the risk of disease and many common complex disorders in humans. A perfectly orchestrated interaction between both nuclear and mitochondrial genomes is essential for mitochondrial biogenesis. In particular, the coordinated expression of many proteins in an extremely complex scenario is required for mtDNA maintenance. Nevertheless, we are still far from identifying all of the factors involved in this process as well as the mechanisms involved in the control of mtDNA levels [37]. The identification of an increasing number of human diseases associated with altered levels of mtDNA has stimulated investigations of the mechanisms and factors involved in the regulation of this trait. In addition, mtDNA levels vary among subjects of different ages and among different tissues within the same individual. These levels are largely influenced by genetic factors, with a heritability (h^2^) ranging between 33% and 65% in different studies [38], [39]. Curran et al. [39] reported a QTL for mtDNA levels on Chromosome 10 (LOD = 3.83) in 1,259 Mexican American subjects from 42 extended families of the Sant Antonio Family Heart Study (SAFHS). They suggested the *mitochondrial transcription factor A* (*TFAM*) and the *translocase of the inner mitochondrial membrane 23* (*TIMM23*) as candidate genes involved in mitochondrial processing for the control of mtDNA levels. In agreement with theses studies, we estimated the h^2^ of mtDNA levels in the Spanish population in 33%. This indicates a substantial genetic component in regulating mtDNA levels and guarantees success in the search for genes that influence this quantitative trait.

The present study aimed to conduct sex-specific genome-wide linkage analyses in a mtDNA-related trait. Accordingly, in our initial linkage scan carried using all of the subjects we found a suggestive linkage signal on Chromosome 2 (LOD = 2.21; p = 7.09e-04). Unlike Curran et al., we did not detect a linkage signal on Chromosome 10. However, when we performed further linkage analyses with subjects according to their sex, we found a strong significant QTL on Chromosome 2 (LOD = 3.09; p = 8.11e-05) and a suggestive QTL on Chromosome 3 (LOD = 2.67; p = 2.27e-04) only in females. Notably, the significant linkage signal from females on Chromosome 2 was at the same region as was the suggestive linkage signal previously detected in the analysis performed using all of the subjects. Interestingly, this genomic region on Chromosome 2 showed no linkage in the analysis performed with males. This demonstrates that the linkage signal detected in the first analysis was the result of the great influence that genes exert over mtDNA levels only in females. In addition, the linkage analysis performed with males only showed a suggestive QTL on Chromosome 1 (LOD = 2.81; p = 1.57e-04). Fine-mapping of these linkage regions revealed significant SNP associations with mtDNA levels. The most significant association was found for the rs10888838 in the linkage region on Chromosome 1 observed in males. This SNP mapped in the *MRPL37* gene, which encodes a component of the mitoribosome involved in the synthesis of key proteins of the OXPHOS system. We noted that this gene was detected exclusively in males through two distinct statistical approaches, such as linkage and association. This combined genetic convergence supports our sex-specific family-based studies to find genes that may differently control quantitative traits of complex diseases according to sex. In addition, these results reinforce the hypothesis that *MRLP37* may contribute to the variation of mtDNA levels only in males. In this sense, functional variants in *MRLP37* having an effect on mtDNA levels needs to be confirmed. The identification of these polymorphisms would be clinically relevant, since it has been reported that a defect in any component of the mitochondrial protein-synthesis machinery might compromise normal mitochondrial function and contribute to disease [40]. On the other hand, fine-mapping of the female-specific linkage region on Chromosome 2 showed a significant association of the rs2140855 with mtDNA levels. However, this SNP association reached significance only in the analysis using all of the subjects. The rs2140855 is intergenic and located ∼170 Kbp downstream of the gene *RNF144* and ∼380 Kbp downstream of the gene *CMPK2. RNF144* encodes an E3 ubiquitin-protein ligase, involved in proteolysis and little information exists with regard this gene and its involvement in disease. However, *CMPK2* deserves special attention, since it is directly related with the mtDNA biology. *CMPK2* encodes a novel mitochondrial kinase that participates in the salvage pathway of deoxyribonucleotide synthesis into the organelle [41]. *CMPK2* also may be involved in the activation of several pyrimidine nucleoside analogs that are widely used as antiviral and anticancer agents, and can cause mtDNA depletion after long term therapy [42]. In addition, mutations in other enzymes of the salvage pathway that are involved in the maintenance of balanced mitochondrial dNTP pools cause severe mitochondrial DNA depletion syndromes [43], [44]. Unfortunately, our genotyping method did not cover the genomic region in *CMPK2*, so no SNP data were available. Nevertheless, the rs2056634 and rs11682769 also reached significance for the association with the phenotype. Interestingly, they were also located around *RNF144* and *CMPK2*. This suggests the existence of a genetic variant in or around this locus that influences the quantity of mtDNA. In keeping with this, 4 SNPs (rs2609101, rs2564023, rs1880800 and rs7571496) are in or around *FLJ41046*, at a distance of ∼700 Kbp upstream of the gene *CMPK2*. With all the above-mentioned, *CMPK2* emerged as a strong candidate gene in the QTL on Chromosome 2 for the control of mtDNA levels. However, we can not rule out other potential candidates genes in this region until functional studies are performed. In addition, the rs813779 and rs2305491 located around *SOX11* and in *TSSC1*, respectively, also were significant in the fine-mapping of the female-specific QTL on Chromosome 2. Notably, *TSSC1* is ∼200 Kbp upstream of the *RNASEH1* gene. *RNASEH1* is also a potential candidate gene in this region. It encodes an endonuclease that specifically degrades the RNA of RNA-DNA hybrids, and it may exert a key function during mtDNA replication [45]. Although all these SNPs are located in the female-specific QTL, it seems that the association with the phenotype occurs when all of the subjects are considered together, but not when males and females are analyzed separately. This observation could be explained by the fact that linkage and association analyses detect different types of genetic variants. In particular, this significant linkage signal could be due to a rare variant present in a group of subjects in these extended pedigrees, whereas the same sample does not provide enough power to detect the effect of the same variant by association. Finally, in the QTL region on Chromosome 3 observed in females, three SNPs showed significant association with mtDNA levels. These polymorphisms were rs2731943 in *VENTXP7*, rs7635386 in *SATB1* and rs161196 in *ZNF659*. *SATB1* encodes a DNA-binding protein involved in transcription regulation, as well as in chromatin organization and nuclear architecture during apoptosis. *VENTXP7* is a non-coding protein gene that is a member of the Vent homeobox gene family. *ZNF659* encodes a nuclear protein involved in nuclear acid binding and zinc ion binding at the transcription level. Although the biological function of these three genes is not directly associated with mtDNA biology, this area deserves further attention given that the linkage and association signals at the same genetic region were significant. Moreover, despite *ZNF659* is located in a female-specific QTL, the rs161196 in this gene showed significant association with mtDNA levels only in the analysis using all of the subjects.

In support of our findings, we detected two haplotypes in the QTL regions on Chromosome 2 and Chromosome 3 in females. These haplotypes have a significant positive effect on mtDNA levels in females only (see File S1, Figure S1, Figure S2 and Figure S3).

Our results emphasize the usefulness of linkage maps in extended pedigrees to detect sex-specific genomic regions for complex traits. This agrees with publications that suggest a strong influence of sex on the susceptibility to common complex diseases in humans [46]. Accordingly, the genetic architecture differs significantly among subjects according to their sex for a large number of quantitative phenotypes [47]. This is consistent with data from other studies of sex-specific heritabilities of common disease-associated quantitative phenotypes such as the case of six sex-specific obesity-related traits in the Chinese population [48] and a set of polymorphisms that influence plasma fibrinogen concentration in a sex-specific manner [49].

It is important to note that despite the sex-specific genetic architecture found in our study, our results show no significant differences in mtDNA levels between males and females. In fact, sex-specific regulation of mtDNA levels does not necessarily imply different quantity of mtDNA between the two sexes. However, if there were significant variations in mtDNA levels between sexes there would be the need for sex-specific mechanisms controlling mtDNA quantity. Accordingly, and in support of our findings, the study of Curran et al. revealed a significant decrease in mtDNA content in males with respect to females in the Caucasian population [39]. In addition, a case-control study including patients affected by renal cell carcinoma reported lower mtDNA content in lymphocytes in males than in females in both cases and controls [38]. Furthermore, other mitochondrial parameters also showed differences between male and female rats for various tissues, thus supporting the existence of sex-specific mechanisms in the control of mitochondrial biogenesis [50], [51]. This agrees with epidemiological data, that reveal less prevalence of some chronic and neurodegenerative diseases associated with mitochondrial dysfunction and oxidative damage in human females (i.e. Parkinson disease). Accordingly, the mitochondrial effects of estrogens may play a role in breast cancer, cardiovascular function, and neuroprotection [52]. Moreover, there is evidence that sex hormones modulate the expression and the activity of proteins that control mitochondrial biogenesis [53], [54]. Thus, estrogens regulate the expression of a number of nuclear- and mitochondrial-encoded genes, which are involved in the control of the OXPHOS system. Also, mitochondria are an important target for the action of estrogens [55]. Accordingly, it is currently well-established that estrogens have direct and indirect effects on mitochondrial activity and biogenesis, but the mechanisms mediating these effects remain to be clarified [56]. Recently, it has been shown that estrogens ameliorate mitochondrial dysfunction in cybrid cells carrying the mtDNA mutation that cause Leber's hereditary optic neuropathy (LHON) and may be responsible for the high prevalence of LHON disease in males [57]. Interestingly, these cybrids were able to increase mtDNA levels after treatment with 17β-estradiol. Consistently with these data, the significant effect of the use of oral contraceptives on mtDNA levels in our sample could be indicative of the control of steroid hormones on mtDNA levels.

Given the growing number of common diseases that are associated with altered mtDNA levels, it has become necessary to investigate the mechanisms that control the quantity of mtDNA in humans. To our knowledge, the present work is the first genome-wide study in extended pedigrees providing data for the existence of sex-specific linkage and association in the control of mtDNA levels. Our results showed a clear sex-specific genetic architecture that may regulate the variation of mtDNA levels in subjects from the GAIT Project. In addition, we provide extensive information regarding potential candidate genes that could modulate mtDNA levels. Our findings may contribute to the understanding of the mechanisms that control mtDNA maintenance. However, these results should be confirmed and extended. Particularly, functional analyses are essential.

## Supporting Information

Figure S1 **Haplotyping analysis in the linkage region detected on Chomosome 2 in females.** Merlin pedigree analysis package was used to perform haplotyping analysis in the family with the maximum contribution to the LOD score in the QTL detected on Chromosome 2 in females (family number 12). Haplotyping analysis in this family revealed the haplotype 5/8/2/8 for the microsatellite genetic polymorphism markers D2S319/D2S2166/D2S2211/D2S162. This haplotype corresponds to the alleles 136/248/249/137 for these markers, respectively; and it was significantly associated with higher levels of mtDNA exclusively in female subjects.
(DOC)

Figure S2 **Haplotyping analysis in the linkage region detected on Chomosome 3 in females.** Merlin pedigree analysis package was used to perform haplotyping analysis in the family with the maximum contribution to the LOD score in the QTL detected on Chromosome 3 in females (family number 12). Haplotyping analysis in this family revealed the haplotype 8/10/12 for the microsatellite markers D3S3589/D3S1263/D3S2338. This haplotype corresponds to the alleles 241/204/114 for these markers, respectively; and it was significantly associated with higher levels of mtDNA exclusively in female subjects.
(DOC)

Figure S3 **Haplotyping analysis in the linkage region detected on Chomosome 1 in males.** Merlin pedigree analysis package was used to perform haplotyping analysis in the family with the maximum contribution to the LOD score in the QTL detected on Chromosome 1 in males. Family number 12 also contributed to the linkage signal identified on Chromosome 1 in males. However, no clear haplotype associated with mtDNA levels was detected in these individuals.
(DOC)

File S1 **Haplotyping Analyses of the Linkage Regions.**
(DOC)

Table S1 **Biological data of the candidate genes proposed for sex-specific variation of mtDNA levels in the GAIT sample.**
(DOC)
