# Supplementary figures and images for: Sex-Specific Regulation of Mitochondrial DNA Levels: Genome-Wide Linkage Analysis to Identify Quantitative Trait Loci
Source: PLoS One. 2012 Aug 20;7(8):e42711. doi: 10.1371/journal.pone.0042711 (PMC3423410; doi:10.1371/journal.pone.0042711)

Figure S1.


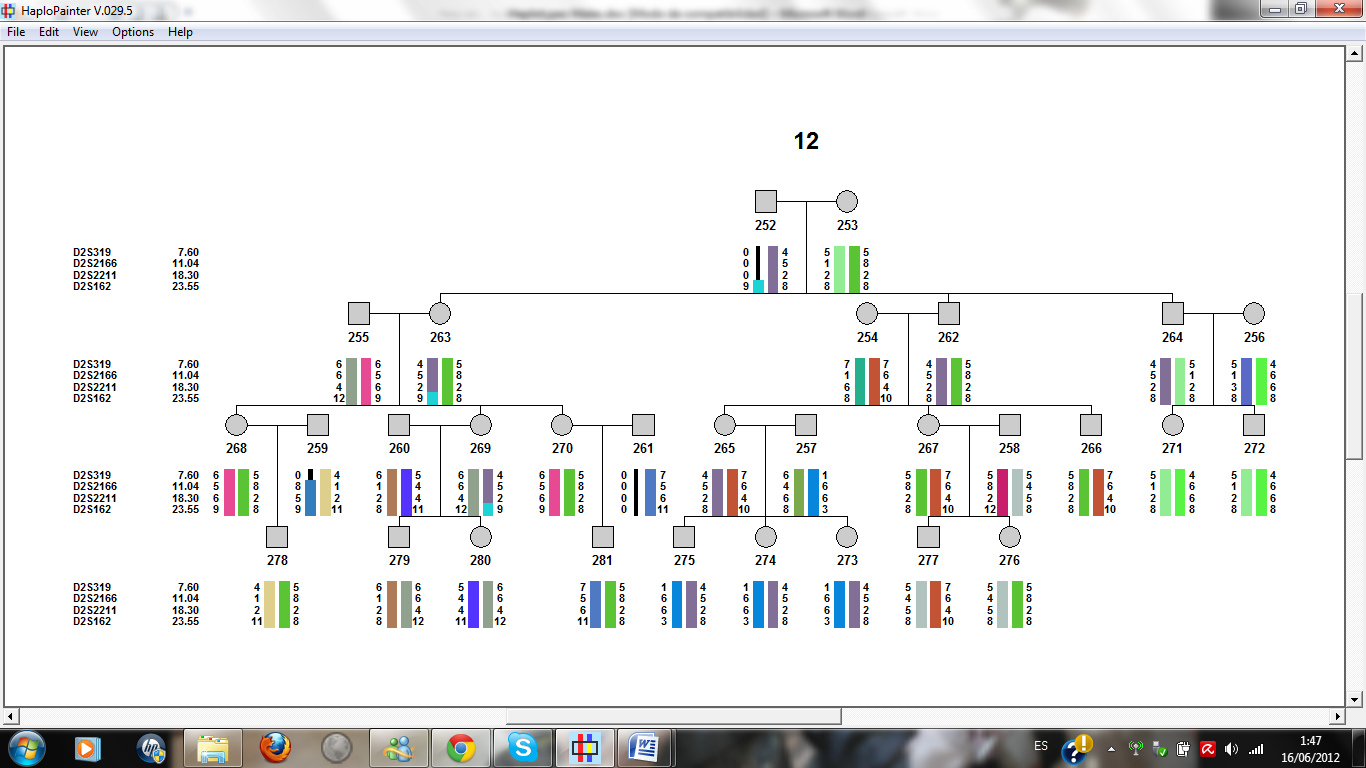

Supplement: Figure S1 — Haplotyping analysis in the linkage region detected on Chomosome 2 in females. Merlin pedigree analysis package was used to perform haplotyping analysis in the family with the maximum contribution to the LOD score in the QTL detected on Chromosome 2 in females (family number 12). Haplotyping analysis in this family revealed the haplotype 5/8/2/8 for the microsatellite genetic polymorphism markers D2S319/D2S2166/D2S2211/D2S162. This haplotype corresponds to the alleles 136/248/249/137 for these markers, respectively; and it was significantly associated with higher levels of mtDNA exclusively in female subjects. (DOC) [file pone.0042711.s001.doc]

Figure S2.


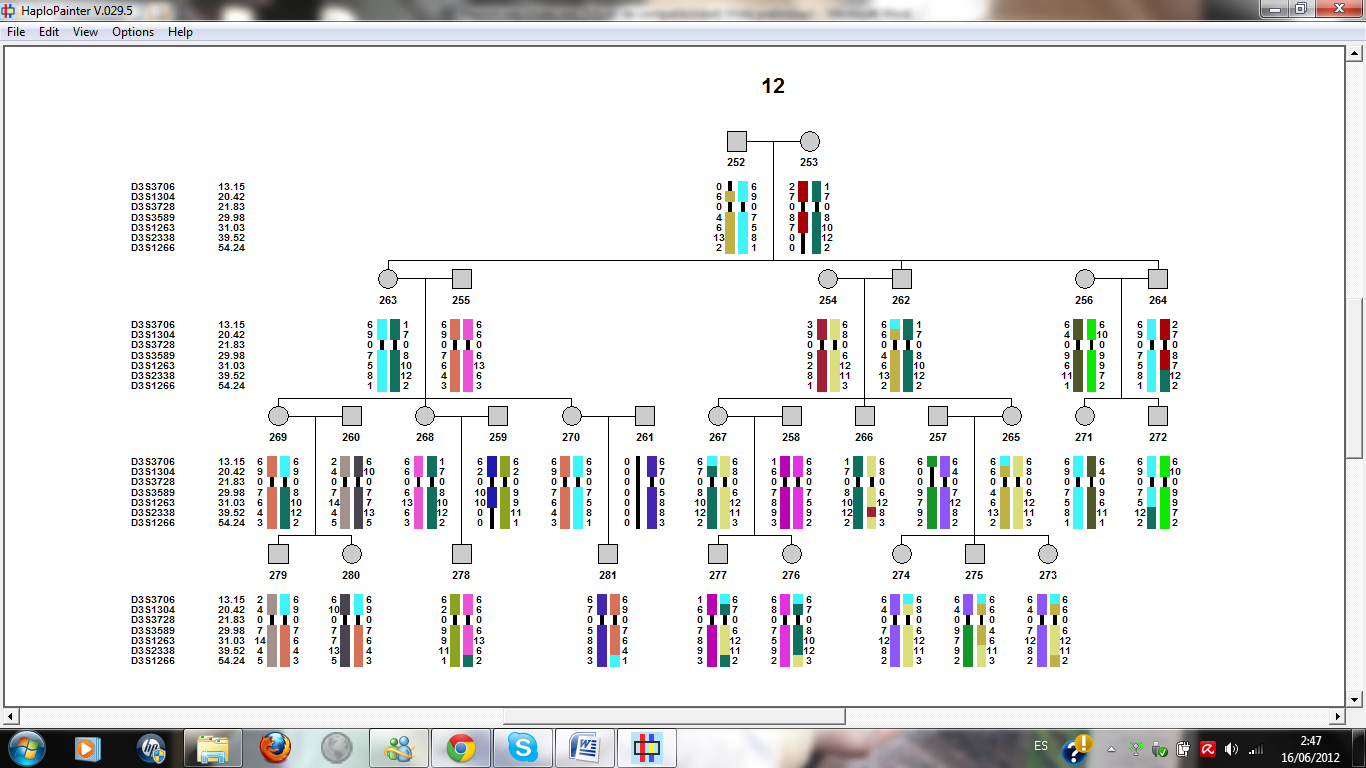

Supplement: Figure S2 — Haplotyping analysis in the linkage region detected on Chomosome 3 in females. Merlin pedigree analysis package was used to perform haplotyping analysis in the family with the maximum contribution to the LOD score in the QTL detected on Chromosome 3 in females (family number 12). Haplotyping analysis in this family revealed the haplotype 8/10/12 for the microsatellite markers D3S3589/D3S1263/D3S2338. This haplotype corresponds to the alleles 241/204/114 for these markers, respectively; and it was significantly associated with higher levels of mtDNA exclusively in female subjects. (DOC) [file pone.0042711.s002.doc]

Figure S3.


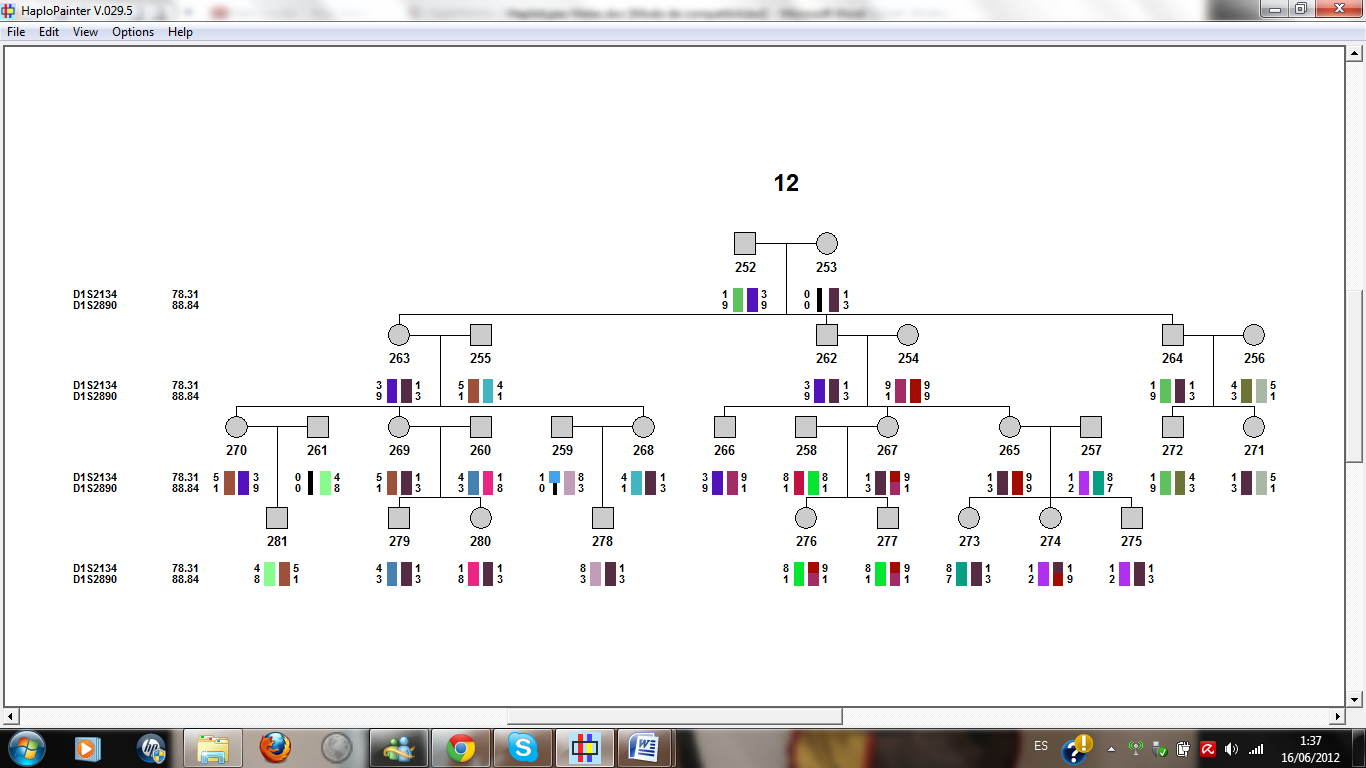

Supplement: Figure S3 — Haplotyping analysis in the linkage region detected on Chomosome 1 in males. Merlin pedigree analysis package was used to perform haplotyping analysis in the family with the maximum contribution to the LOD score in the QTL detected on Chromosome 1 in males. Family number 12 also contributed to the linkage signal identified on Chromosome 1 in males. However, no clear haplotype associated with mtDNA levels was detected in these individuals. (DOC) [file pone.0042711.s003.doc]
