# Supplementary material for: Sex-Specific Regulation of Mitochondrial DNA Levels: Genome-Wide Linkage Analysis to Identify Quantitative Trait Loci
Source: PLoS One. 2012 Aug 20;7(8):e42711. doi: 10.1371/journal.pone.0042711 (PMC3423410; doi:10.1371/journal.pone.0042711)
Supplement: File S1 — Haplotyping Analyses of the Linkage Regions. (DOC) [file pone.0042711.s004.doc]

**Haplotyping Analyses of the Linkage Regions**

We aimed to perform haplotyping analyses in the linkage regions detected in our study on Chomosome 2 and Chromosome 3 in females and on Chromosome 1 in males. The identification of haplotypes in these regions may support our results of sex-specificity with regard to mtDNA levels. In these sense, the mtDNA levels of the members with the same haplotype and sex from the same family should be similar. We have used the multipoint engine for rapid likelihood inference (Merlin) pedigree analysis package to perform haplotyping analysis in the family with the maximum contribution to the LOD score in each QTL [1]. Figures for pedigrees showing the haplotypes from Merlin were performed by the software HaploPainter v.029.5.

The results of the analyses are as follows:

Family number 12 strongly contributed to the linkage signal identified on Chromosome 2 in females only.

Haplotyping analysis with Merlin in this family revealed the haplotype 5 / 8 / 2 / 8 for the microsatellite genetic polymorphism markers D2S319 / D2S2166 / D2S2211 / D2S162 (Figure S1). This haplotype corresponds to the alleles 136 / 248 / 249 / 137 for these markers, respectively.

To analyze the effect of the haplotype 5 / 8 / 2 / 8 on mtDNA levels according to the sex of the subjects we compared the median values of mtDNA levels (±SD) between carriers and non-carriers subjects. Females carrying the haplotype exhibited significantly higher mtDNA levels than females not carrying the haplotype (0.359±0.089 vs 0.224±0.104, respectively; p<0.05). This result indicates that this haplotype have a significant positive effect on mtDNA levels in females.

In addition, the comparison between all of the subjects carrying the haplotype and non-carriers subjects, independently on the sex, showed not significant differences (0.312±0.103 vs 0.308±0.149; p=NS). This clearly demonstrates that this haplotype have an effect only in females.

Family number 12 strongly contributed to the linkage signal identified on Chromosome 3 in females only.

Haplotyping analysis with Merlin in this family revealed the haplotype 8 / 10 / 12 for the microsatellite markers D3S3589 / D3S1263 / D3S2338 (Figure S2). This haplotype corresponds to the alleles 241 / 204 / 114 for these markers, respectively.

We compared the median values of mtDNA levels (±SD) between carriers and non-carriers subjects to analyze the effect of the haplotype 8 / 10 / 12 on mtDNA levels according to the sex of the subjects. Females carrying this haplotype exhibited significantly higher mtDNA levels than females not carrying the haplotype (0.361±0.087 vs 0.223±0.111, respectively; p<0.05). This result indicates that this haplotype have a significant positive effect on mtDNA levels in females.

In addition, the comparison between all of the subjects carrying the haplotype and non-carriers subjects, independently on the sex, showed not significant differences (0.315±0.119 vs 0.307±0.134; p=NS). This clearly demonstrates that this haplotype have an effect only in females.

Family number 12 also contributed to the linkage signal identified on Chromosome 1 in males only. However, we were not able to detect a clear haplotype associated with the phenotype in these individuals (Figure S3).

In conclusion, the haplotyping analyses performed in the two female-specific QTLs identified on Chromosome 2 and Chromosome 3 revealed two haplotypes significantly associated with higher levels of mtDNA exclusively in female subjects. These results clearly support our findings of sex contribution to mtDNA levels. However, we failed to identify haplotypes in the male-specific QTL on Chromosome 1, probably because of sample size.

**REFERENCES**

1. Abecasis GR, Cherny SS, Cookson WO, Cardon LR (2002) Merlin--rapid analysis of dense genetic maps using sparse gene flow trees. Nat Genet 30: 97-101.
