# Supplementary material for: Sex-Specific Regulation of Mitochondrial DNA Levels: Genome-Wide Linkage Analysis to Identify Quantitative Trait Loci
Source: PLoS One. 2012 Aug 20;7(8):e42711. doi: 10.1371/journal.pone.0042711 (PMC3423410; doi:10.1371/journal.pone.0042711)
Supplement: Table S1 — Biological data of the candidate genes proposed for sex-specific variation of mtDNA levels in the GAIT sample. (DOC) [file pone.0042711.s005.doc]

Table S1. Biological data of the candidate genes proposed for sex-specific variation of mtDNA levels in the GAIT sample.

| Gene symbol | Chr. | Sex-specificity | Biological remarks |
| --- | --- | --- | --- |
| *CMPK2* | 2 | Females | - mitochondrial kinase recently identified that participates in the salvage pathway for pyrimidine biosynthesis [1]. - involved in the activation of several pyrimidine nucleoside analogs used as antiviral and anticancer agents that cause mtDNA depletion after long term therapy [2]. - mutations in other enzymes of the salvage pathway (dGK and TK2) cause severe mtDNA depletion syndromes [3,4]. - may be involved in maintaining a balanced supply of dNTPs to warrant mtDNA replication [5]. |
| *RNASEH1* | 2 | Females | - participates in RNA degradation from RNA-DNA hybrids that are RNA primers of DNA replication and allow proper formation of mature DNA [6]. - mainly present in nuclei but has also been targeted to mitochondria [7,8]. - *RNASEH1* knock-out mice produce a decrease of total DNA, but a higher relative decrease of mtDNA levels in a particular stage when embryos begin to depend on oxidative phosphorylation for energy production [9]. This suggests the importance of the *RNASEH1* in the control of mtDNA levels in mammals. |
| *MRPS25* | 3 | Females | - takes part of the mitoribosome small subunit 28S involed in protein translation into the organelle. - mitoribosomes are essential for energy production and have been associated with mitochondrial disease [10]. - genetic variation in *MRPS25* has been linked to dilated cardiomyopathy [11]. |
| *TIMM40* | 3 | Females | - essential protein of the recently identified redox-dependent MIA pathway that specifically transports mitochondrial precursor proteins to the IMS [12]. - its oxidation allows the release of the protein precursors to the IMS and the electrons delivered are transferred to the cyt c of the MRC [12]. - mutant yeast fail to import several small essential IMS proteins that forms mature TIM complexes [13]. - responsible of the import and distribution of the Sod1 in the IMS to scavenge ROS in yeast [14]. This is of interest in amyotrophic lateral sclerosis. |
| *C3orf31* | 3 | Females | - mitochondrial matrix protein peripherally associated with the inner membrane, involved in protein import into the organelle. Maintains the functional integrity of the TIM23 protein translocator complex [15]. - essential for an early step of cardiolipin (CL) biosynthesis, as it might be specifically required for the import of intermediate proteins necessary for this process [16,17]. - abnormal CL density in the mitochondrial inner membrane can alter enzyme activity of the MRC and favour electron leak from the organelle and ROS generation [18]. - MRC defects and loss of mtDNA have been associated with the lack of CL in yeast [19]. |
| *PPARG* | 3 | Females | - isoform γ of the nuclear receptor superfamily of transcription factors that originally regulate adipocyte differentiation and glucose homeostasis [20]. - recently attributed pleiotropic effects. Evidence for a role in various diseases based on its anti-inflammatory, cell cycle arresting, and proapoptotic properties [21]. - some of the disorders associated with PPARγ pathway are sepsis and inflammation [22,23] as well as cardiovascular disease [24,25]. - interacts with co-activators, including PGC-1α and SRC-1. - promotes mitochondrial biogenesis in different tissues through the interaction with PGC-1α [26]. It has been associated with neuroprotection [27,28,29], as PPARγ agonists up-regulate key genes of mitochondrial biogenesis and increase mtDNA levels [30], and the response to antidiabetic drugs [31,32]. - its genetic variance may influence body mass index [33], carotid intimal medial thickness (a measure of atherosclerosis) [34] and susceptibility to glioma type 1 [35]. - defects in PPARγ have been associated with numerous diseases including obesity [36,37], diabetes [38], atherosclerosis [34], colon cancer [39], hypertension [38], and familial partial lipodystrophy [40]. |
| *OGG1* | 3 | Females | - 8-oxoguanine (8-oxodG) DNA glycosylase/apurinic DNA lyase of the base excision repair pathway. - removes 8-oxodG residues from DNA, which are the major products of ROS-derived DNA oxidation. - alternative splicing generates the nuclear isoform *α-OGG1* and the mitochondrial isoform *β-OGG1* [41]. - 8-oxodG accumulates in mtDNA at levels three-fold higher than in nuclear DNA, so OGG1 repair activity is particularly important in mtDNA [41,42]. - 8-oxodG is highly mutagenic, leading to GC to TA transversions in DNA, which has been related to carcinogenesis [43,44]. A somatic mutation in *OGG1* interferes with the mitochondrial localization of the *β-OGG1* isoform in kidney cancer in humans [45]. - highly polymorphic with some sequence variations or mutations correlating with low repair activity of the protein and the risk for cancers [46,47]. |
| *MRPL37* | 1 | Males | - takes part of the mitoribosome large subunit 39S involed in protein translation into the organelle. - mitoribosomes are essential for energy production and they have been associated with mitochondrial disease [10]. - genetic variation in *MRPL37* has been related to PD [11]. |
| *PARS2* | 1 | Males | - aminoacyl-tRNA synthetase involved in protein biosynthesis into the organelle. - defects in any component of the mitochondrial protein-synthesis machinery might compromise normal mitochondrial function and cause disease [48]. |
| *ATPAF1* | 1 | Males | - plays an essential role for the assembly of the mitochondrial subunits F1-F0 of the ATP synthase (CV) of the OXPHOS system. - correct assembly of F1-F0 is critical for ATP generation. ATPAF1 interacts with the protein subunit ATP5B for normal CV activity. - low expression levels of ATP5B and mitochondrial dysfunction have been associated with decreased mtDNA levels, as well as disease progression and poor outcome in colorectal cancer patients [49]. |
| *CPT2* | 1 | Males | - mitochondrial protein of the carnitine shuttle that allows the entry of cytosolic long-chain fatty acids to the mitochondrial matrix for β-oxidation. - defects in *CPT2* are the cause of carnitine palmitoyltransferase 2 deficiency (CPT2D), a late-onset autosomal recessive disorder [50]. - no data about mtDNA levels regarding CPT2D. |
| *UQCRH* | 1 | Males | - ubiquinol-cyt c reductase hinge protein subunit of CIII of the MRC, essential for the formation of the cyt c1-c complex. - may regulate the electron transfer through CIII, as well as mitochondrial respiration and the release of cyt c from mitochondria to the cytosol to induce apoptosis [51]. - UQCRH protein is modified by oxidative damage in murine hearts infected by *Trypanosoma cruzi*, which cause chagasic cardiomyopathy associated with mitochondrial dysfunction and reduced ATP synthesis in the myocardium. The extent of the oxidative damage usually correlates with the impaired mitochondrial parameters [52]. - low expression has been associated with PD [53] as well as insulin resistance and type 2 diabetes [54,55]. - elevated expression has been observed *in vitro* in cancerous cells, thus supporting previous observation that neoplasia favors the induction of OXPHOS genes, as a response to a higher energy demand [56]. - downregulation by either translocation or inactivation by methylation has been shown in some cancerous cells [57]. *UQCRH* might influence tumorigenesis through superoxide overproduction, which may activate transcription of nuclear genes involved in cell proliferation and lead to tumor formation. - plays a central role in the effects of a calorie restriction regimen on life extension in mice, which is thought that it might be a consequence of less free radicals production contributing to lifespan [58]. |
| *CMPK1* | 1 | Males | - cytosolic kinase involved in the pyrimidine biosynthesis [59]. - contributes to the maintenance of balanced cellular dNTP pools, required for accurate DNA synthesis. Depletion of mtDNA may be the consequence of imbalanced dNTP pools in many human diseases [60]. - cytosolic and mitochondrial cross-talk allows the two compartments to exchange pool components across the inner mitochondrial membrane. Thus, regulation of dNTP pools in the organelle depends on the mitochondrial membrane carriers of dNTPs, as well as on the cytosolic and mitochondrial enzymes involved in nucleotide synthesis. |

Chr.: Chromosome; dGK: deoxyguanosine kinase; TK2: thymidine kinase 2; dNTPs: deoxyribonucleoside triphosphates; MIA: mitochondrial IMS (intermembrane space) import and assembly; cyt c: cytochrome c; MRC: mitochondrial respiratory chain; TIM: translocase of the inner membrane; Sod1: copper-zinc superoxid dismutase; ROS: reactive oxygen especies; PGC-1α: PPARγ coactivator 1α; SRC-1: steroid receptor co-activating factor-1; PD: Parkinson disease; CV: complex V; OXPHOS system: oxidative phosphorilation system; CIII: complex III.

**REFERENCES**

1. Xu Y, Johansson M, Karlsson A (2008) Human UMP-CMP kinase 2, a novel nucleoside monophosphate kinase localized in mitochondria. J Biol Chem 283: 1563-1571.

2. Dalakas MC, Semino-Mora C, Leon-Monzon M (2001) Mitochondrial alterations with mitochondrial DNA depletion in the nerves of AIDS patients with peripheral neuropathy induced by 2'3'-dideoxycytidine (ddC). Lab Invest 81: 1537-1544.

3. Marti R, Nascimento A, Colomer J, Lara MC, Lopez-Gallardo E, et al. (2010) Hearing loss in a patient with the myopathic form of mitochondrial DNA depletion syndrome and a novel mutation in the TK2 gene. Pediatr Res 68: 151-154.

4. Freisinger P, Futterer N, Lankes E, Gempel K, Berger TM, et al. (2006) Hepatocerebral mitochondrial DNA depletion syndrome caused by deoxyguanosine kinase (DGUOK) mutations. Arch Neurol 63: 1129-1134.

5. Rampazzo C, Miazzi C, Franzolin E, Pontarin G, Ferraro P, et al. (2010) Regulation by degradation, a cellular defense against deoxyribonucleotide pool imbalances. Mutat Res 703: 2-10.

6. Cerritelli SM, Crouch RJ (2009) Ribonuclease H: the enzymes in eukaryotes. FEBS J 276: 1494-1505.

7. Pileur F, Toulme JJ, Cazenave C (2000) Eukaryotic ribonucleases HI and HII generate characteristic hydrolytic patterns on DNA-RNA hybrids: further evidence that mitochondrial RNase H is an RNase HII. Nucleic Acids Res 28: 3674-3683.

8. Engel ML, Hines JC, Ray DS (2001) The Crithidia fasciculata RNH1 gene encodes both nuclear and mitochondrial isoforms of RNase H. Nucleic Acids Res 29: 725-731.

9. Cerritelli SM, Frolova EG, Feng C, Grinberg A, Love PE, et al. (2003) Failure to produce mitochondrial DNA results in embryonic lethality in Rnaseh1 null mice. Mol Cell 11: 807-815.

10. O'Brien TW, O'Brien BJ, Norman RA (2005) Nuclear MRP genes and mitochondrial disease. Gene 354: 147-151.

11. Sylvester JE, Fischel-Ghodsian N, Mougey EB, O'Brien TW (2004) Mitochondrial ribosomal proteins: candidate genes for mitochondrial disease. Genet Med 6: 73-80.

12. Riemer J, Fischer M, Herrmann JM (2011) Oxidation-driven protein import into mitochondria: Insights and blind spots. Biochim Biophys Acta 1808: 981-989.

13. Chacinska A, Pfannschmidt S, Wiedemann N, Kozjak V, Sanjuan Szklarz LK, et al. (2004) Essential role of Mia40 in import and assembly of mitochondrial intermembrane space proteins. EMBO J 23: 3735-3746.

14. Kloppel C, Michels C, Zimmer J, Herrmann JM, Riemer J (2010) In yeast redistribution of Sod1 to the mitochondrial intermembrane space provides protection against respiration derived oxidative stress. Biochem Biophys Res Commun 403: 114-119.

15. Tamura Y, Harada Y, Yamano K, Watanabe K, Ishikawa D, et al. (2006) Identification of Tam41 maintaining integrity of the TIM23 protein translocator complex in mitochondria. J Cell Biol 174: 631-637.

16. Osman C, Haag M, Wieland FT, Brugger B, Langer T (2010) A mitochondrial phosphatase required for cardiolipin biosynthesis: the PGP phosphatase Gep4. EMBO J 29: 1976-1987.

17. Kutik S, Rissler M, Guan XL, Guiard B, Shui G, et al. (2008) The translocator maintenance protein Tam41 is required for mitochondrial cardiolipin biosynthesis. J Cell Biol 183: 1213-1221.

18. Pfeiffer K, Gohil V, Stuart RA, Hunte C, Brandt U, et al. (2003) Cardiolipin stabilizes respiratory chain supercomplexes. J Biol Chem 278: 52873-52880.

19. Zhong Q, Gohil VM, Ma L, Greenberg ML (2004) Absence of cardiolipin results in temperature sensitivity, respiratory defects, and mitochondrial DNA instability independent of pet56. J Biol Chem 279: 32294-32300.

20. Mukherjee R, Jow L, Croston GE, Paterniti JR, Jr. (1997) Identification, characterization, and tissue distribution of human peroxisome proliferator-activated receptor (PPAR) isoforms PPARgamma2 versus PPARgamma1 and activation with retinoid X receptor agonists and antagonists. J Biol Chem 272: 8071-8076.

21. Schmidt MV, Brune B, von Knethen A (2010) The nuclear hormone receptor PPARgamma as a therapeutic target in major diseases. ScientificWorldJournal 10: 2181-2197.

22. Zingarelli B, Cook JA (2005) Peroxisome proliferator-activated receptor-gamma is a new therapeutic target in sepsis and inflammation. Shock 23: 393-399.

23. Martin H (2010) Role of PPAR-gamma in inflammation. Prospects for therapeutic intervention by food components. Mutat Res 690: 57-63.

24. van Bilsen M, van Nieuwenhoven FA (2010) PPARs as therapeutic targets in cardiovascular disease. Expert Opin Ther Targets 14: 1029-1045.

25. Touyz RM, Schiffrin EL (2006) Peroxisome proliferator-activated receptors in vascular biology-molecular mechanisms and clinical implications. Vascul Pharmacol 45: 19-28.

26. Puigserver P (2005) Tissue-specific regulation of metabolic pathways through the transcriptional coactivator PGC1-alpha. Int J Obes (Lond) 29 Suppl 1: S5-9.

27. Kiaei M, Kipiani K, Chen J, Calingasan NY, Beal MF (2005) Peroxisome proliferator-activated receptor-gamma agonist extends survival in transgenic mouse model of amyotrophic lateral sclerosis. Exp Neurol 191: 331-336.

28. Breidert T, Callebert J, Heneka MT, Landreth G, Launay JM, et al. (2002) Protective action of the peroxisome proliferator-activated receptor-gamma agonist pioglitazone in a mouse model of Parkinson's disease. J Neurochem 82: 615-624.

29. Aoun P, Watson DG, Simpkins JW (2003) Neuroprotective effects of PPARgamma agonists against oxidative insults in HT-22 cells. Eur J Pharmacol 472: 65-71.

30. Miglio G, Rosa AC, Rattazzi L, Collino M, Lombardi G, et al. (2009) PPARgamma stimulation promotes mitochondrial biogenesis and prevents glucose deprivation-induced neuronal cell loss. Neurochem Int 55: 496-504.

31. Wilson-Fritch L, Nicoloro S, Chouinard M, Lazar MA, Chui PC, et al. (2004) Mitochondrial remodeling in adipose tissue associated with obesity and treatment with rosiglitazone. J Clin Invest 114: 1281-1289.

32. Rong JX, Qiu Y, Hansen MK, Zhu L, Zhang V, et al. (2007) Adipose mitochondrial biogenesis is suppressed in db/db and high-fat diet-fed mice and improved by rosiglitazone. Diabetes 56: 1751-1760.

33. Masud S, Ye S (2003) Effect of the peroxisome proliferator activated receptor-gamma gene Pro12Ala variant on body mass index: a meta-analysis. J Med Genet 40: 773-780.

34. Temelkova-Kurktschiev T, Hanefeld M, Chinetti G, Zawadzki C, Haulon S, et al. (2004) Ala12Ala genotype of the peroxisome proliferator-activated receptor gamma2 protects against atherosclerosis. J Clin Endocrinol Metab 89: 4238-4242.

35. Zhou XP, Smith WM, Gimm O, Mueller E, Gao X, et al. (2000) Over-representation of PPARgamma sequence variants in sporadic cases of glioblastoma multiforme: preliminary evidence for common low penetrance modifiers for brain tumour risk in the general population. J Med Genet 37: 410-414.

36. Valve R, Sivenius K, Miettinen R, Pihlajamaki J, Rissanen A, et al. (1999) Two polymorphisms in the peroxisome proliferator-activated receptor-gamma gene are associated with severe overweight among obese women. J Clin Endocrinol Metab 84: 3708-3712.

37. Ristow M, Muller-Wieland D, Pfeiffer A, Krone W, Kahn CR (1998) Obesity associated with a mutation in a genetic regulator of adipocyte differentiation. N Engl J Med 339: 953-959.

38. Barroso I, Gurnell M, Crowley VE, Agostini M, Schwabe JW, et al. (1999) Dominant negative mutations in human PPARgamma associated with severe insulin resistance, diabetes mellitus and hypertension. Nature 402: 880-883.

39. Sarraf P, Mueller E, Smith WM, Wright HM, Kum JB, et al. (1999) Loss-of-function mutations in PPAR gamma associated with human colon cancer. Mol Cell 3: 799-804.

40. Agarwal AK, Garg A (2002) A novel heterozygous mutation in peroxisome proliferator-activated receptor-gamma gene in a patient with familial partial lipodystrophy. J Clin Endocrinol Metab 87: 408-411.

41. Boiteux S, Radicella JP (2000) The human OGG1 gene: structure, functions, and its implication in the process of carcinogenesis. Arch Biochem Biophys 377: 1-8.

42. de Souza-Pinto NC, Eide L, Hogue BA, Thybo T, Stevnsner T, et al. (2001) Repair of 8-oxodeoxyguanosine lesions in mitochondrial dna depends on the oxoguanine dna glycosylase (OGG1) gene and 8-oxoguanine accumulates in the mitochondrial dna of OGG1-defective mice. Cancer Res 61: 5378-5381.

43. Shinmura K, Yokota J (2001) The OGG1 gene encodes a repair enzyme for oxidatively damaged DNA and is involved in human carcinogenesis. Antioxid Redox Signal 3: 597-609.

44. Chevillard S, Radicella JP, Levalois C, Lebeau J, Poupon MF, et al. (1998) Mutations in OGG1, a gene involved in the repair of oxidative DNA damage, are found in human lung and kidney tumours. Oncogene 16: 3083-3086.

45. Audebert M, Charbonnier JB, Boiteux S, Radicella JP (2002) Mitochondrial targeting of human 8-oxoguanine DNA glycosylase hOGG1 is impaired by a somatic mutation found in kidney cancer. DNA Repair (Amst) 1: 497-505.

46. Shinmura K, Yamaguchi S, Saitoh T, Kohno T, Yokota J (2001) Somatic mutations and single nucleotide polymorphisms of base excision repair genes involved in the repair of 8-hydroxyguanine in damaged DNA. Cancer Lett 166: 65-69.

47. Hung RJ, Hall J, Brennan P, Boffetta P (2005) Genetic polymorphisms in the base excision repair pathway and cancer risk: a HuGE review. Am J Epidemiol 162: 925-942.

48. Jacobs HT, Turnbull DM (2005) Nuclear genes and mitochondrial translation: a new class of genetic disease. Trends Genet 21: 312-314.

49. Lin PC, Lin JK, Yang SH, Wang HS, Li AF, et al. (2008) Expression of beta-F1-ATPase and mitochondrial transcription factor A and the change in mitochondrial DNA content in colorectal cancer: clinical data analysis and evidence from an in vitro study. Int J Colorectal Dis 23: 1223-1232.

50. Sigauke E, Rakheja D, Kitson K, Bennett MJ (2003) Carnitine palmitoyltransferase II deficiency: a clinical, biochemical, and molecular review. Lab Invest 83: 1543-1554.

51. Okazaki M, Ishibashi Y, Asoh S, Ohta S (1998) Overexpressed mitochondrial hinge protein, a cytochrome c-binding protein, accelerates apoptosis by enhancing the release of cytochrome c from mitochondria. Biochem Biophys Res Commun 243: 131-136.

52. Wen JJ, Garg N (2004) Oxidative modification of mitochondrial respiratory complexes in response to the stress of Trypanosoma cruzi infection. Free Radic Biol Med 37: 2072-2081.

53. Hauser MA, Li YJ, Xu H, Noureddine MA, Shao YS, et al. (2005) Expression profiling of substantia nigra in Parkinson disease, progressive supranuclear palsy, and frontotemporal dementia with parkinsonism. Arch Neurol 62: 917-921.

54. Patti ME, Butte AJ, Crunkhorn S, Cusi K, Berria R, et al. (2003) Coordinated reduction of genes of oxidative metabolism in humans with insulin resistance and diabetes: Potential role of PGC1 and NRF1. Proc Natl Acad Sci U S A 100: 8466-8471.

55. Lu H, Yang Y, Allister EM, Wijesekara N, Wheeler MB (2008) The identification of potential factors associated with the development of type 2 diabetes: a quantitative proteomics approach. Mol Cell Proteomics 7: 1434-1451.

56. Liu AY, Bradner RC (1993) Elevated expression of the human mitochondrial hinge protein gene in cancer. Cancer Res 53: 2460-2465.

57. Modena P, Testi MA, Facchinetti F, Mezzanzanica D, Radice MT, et al. (2003) UQCRH gene encoding mitochondrial Hinge protein is interrupted by a translocation in a soft-tissue sarcoma and epigenetically inactivated in some cancer cell lines. Oncogene 22: 4586-4593.

58. Goertzel B, Pennachin C, de Alvarenga Mudado M, de Souza Coelho L (2008) Identifying the genes and genetic interrelationships underlying the impact of calorie restriction on maximum lifespan: an artificial intelligence-based approach. Rejuvenation Res 11: 735-748.

59. Liou JY, Dutschman GE, Lam W, Jiang Z, Cheng YC (2002) Characterization of human UMP/CMP kinase and its phosphorylation of D- and L-form deoxycytidine analogue monophosphates. Cancer Res 62: 1624-1631.

60. Ashley N, Adams S, Slama A, Zeviani M, Suomalainen A, et al. (2007) Defects in maintenance of mitochondrial DNA are associated with intramitochondrial nucleotide imbalances. Hum Mol Genet 16: 1400-1411.
